# Beyond DCFH-DA: A Critical Review of Hydrogen Peroxide and Superoxide Detection Strategies in Mammalian Living Systems (2015–2026)

**DOI:** 10.3390/ijms27156912

**Published:** 2026-08-01

**Authors:** Luciana Alexandra Pavelescu, Antoanela Curici, Violeta Liuba Călin

**Affiliations:** 1 Department of Cellular and Molecular Biology and Histology, “Carol Davila” University of Medicine and Pharmacy, 8 Eroii Sanitari Boulevard, 050474 Bucharest, Romania; luciana.pavelescu@umfcd.ro; 2 Synevo Romania, 021408 Bucharest, Romania; 3 Biophysics Department, Faculty of Medicine, “Carol Davila” University of Medicine and Pharmacy, 8 Eroii Sanitari Boulevard, 050474 Bucharest, Romania; violeta.calin@umfcd.ro

**Keywords:** reactive oxygen species, hydrogen peroxide, fluorescent probes, HyPer biosensor, roGFP, electron paramagnetic resonance, mass spectrometry, redox biology, oxidative eustress, nanosensors

## Abstract

Reactive oxygen species (ROS) regulate cellular signaling at physiological concentrations and drive tissue damage when their generation exceeds antioxidant defenses. The conceptual reframing of the field into oxidative eustress (low, controlled redox signaling) and oxidative distress (supraphysiological levels causing biomolecular damage), alongside parallel advances in detection chemistry and genetically encoded biosensors, has transformed how investigators measure ROS in living systems. This review provides a critical, methods-focused update covering the contemporary toolkit, with particular emphasis on advances from 2015 to 2026 while incorporating earlier foundational work where it remains indispensable to interpretation. Consistent with the title and reflecting both the maturity of the available chemistry and the weight of the recent literature, our emphasis falls on hydrogen peroxide and mammalian experimental systems; superoxide, the hydroxyl radical, and singlet oxygen are addressed primarily where their detection intersects with the platforms reviewed here, and non-mammalian models are considered only selectively. Readers seeking dedicated coverage of these other species or of plant, microbial, and invertebrate systems are directed to the specialized reviews cited throughout. Five complementary measurement platforms are evaluated: (i) electron paramagnetic resonance spectroscopy with classical nitrone spin traps and the newer cyclic hydroxylamine probes; (ii) small-molecule fluorescent probes, with particular emphasis on the boronate-based, activity-based sensing platform that has supplanted 2′,7′-dichlorofluorescin diacetate for hydrogen peroxide imaging; (iii) genetically encoded biosensors of the HyPer and roGFP families, which now permit ratiometric, organelle-resolved, and longitudinal measurements; (iv) mass-spectrometry-based quantification of oxidation products and radical adducts, including isoprostanes, 2-hydroxyethidium, and redox-modified cysteines via chemical proteomics; and (v) electrochemical and nanosensor approaches enabling real-time single-cell measurements. The selectivity, sensitivity, temporal resolution, spatial resolution, and quantitative capability of each platform are critically compared. Reliance on a single non-specific probe is no longer sufficient as the sole evidence base for quantitative or species-specific claims; contemporary investigators are expected to apply complementary approaches and to validate findings across modalities. Standardization of reporting, integration with single-cell omics, and clinical translation of validated mass-spectrometry biomarkers are identified as priorities for the coming decade.

## 1. Introduction

Reactive oxygen species (ROS) are oxygen-containing molecules of high chemical reactivity that originate from the partial reduction in molecular oxygen during aerobic metabolism. The category encompasses both free radicals—species with one or more unpaired electrons in their outer orbitals, including superoxide (O_2_^•−^), hydroxyl (•OH), and peroxyl (ROO•) radicals—and non-radical oxidants such as hydrogen peroxide (H_2_O_2_), singlet oxygen (^1^O_2_), and hypochlorous acid (HOCl) [1,2]. When closely related reactive nitrogen species (RNS), such as nitric oxide (NO•) and peroxynitrite (ONOO−), are considered together with ROS, the more inclusive term reactive oxygen and nitrogen species (RONS) is preferred [3].

A decade ago, when the methodological landscape of ROS detection in living systems was last reviewed for the broader biomedical readership [4], the field was dominated by a handful of fluorescent probes—most prominently 2′,7′-dichlorofluorescin diacetate (DCFH-DA)—together with thiobarbituric acid reactive substances assays and electron paramagnetic resonance (EPR) spin trapping. Since then, three concurrent developments have reshaped the discipline. First, the conceptual reframing of oxidative stress by Sies and colleagues into the complementary states of oxidative eustress (low, controlled fluxes of H_2_O_2_ that drive physiological signaling) and oxidative distress (supraphysiological levels causing biomolecular damage) [5,6,7] has demanded measurement tools capable of resolving nanomolar concentration differences between cellular compartments. Second, the development of activity-based fluorescent probes—particularly the boronate-based family pioneered by the Chang laboratory—has provided chemoselective access to specific ROS, replacing the non-specific oxidation chemistry of older indicators [8,9,10]. Third, genetically encoded biosensors of the HyPer and reduction–oxidation-sensitive green fluorescent protein (roGFP) families have enabled the first reliable measurements of H_2_O_2_ and glutathione redox potential in defined subcellular compartments and intact organisms [11,12,13,14].

These advances have produced biologically meaningful reference values for redox states in living cells. The intracellular steady-state concentration of H_2_O_2_ is now estimated to lie in the 1–10 nM range under physiological conditions, with adaptive responses engaged at approximately 100 nM and biomolecular damage at higher concentrations [6,15,16,17,18]. Mitochondrial matrix concentrations differ from cytosolic ones by orders of magnitude, and individual organelles maintain distinct redox set points modulated by circadian rhythm and exogenous exposures [5,19]. Such resolution was simply not attainable with the earlier generation of probes.

Despite this progress, the published literature continues to be encumbered by an over-reliance on DCFH-DA—a probe that lacks selectivity for H_2_O_2_, is oxidized by multiple species (peroxynitrite, hydroxyl radical, hypochlorite, several heme proteins), produces fluorescent products through autoxidation, and is incompatible with quantitative claims [9,20,21]. The American Heart Association consensus statement explicitly cautions against the interpretation of DCFH-DA fluorescence as a direct readout of H_2_O_2_ [22], and recent expert panels have echoed this position [3,11]. Updating the methodological standards of the broader research community is therefore both timely and necessary.

This review provides a critical, methods-focused update covering: (i) the major sources and chemistry of ROS that any detection method must contend with; (ii) the spectrum of measurement platforms now available—EPR spectroscopy, small-molecule fluorescent probes, genetically encoded biosensors, mass spectrometry, and electrochemical sensors—with critical evaluation of each; (iii) practical guidance on probe selection and validation; and (iv) an assessment of the field’s current limitations and future directions. Particular emphasis is placed on developments from 2015 to 2026, during which the toolkit has matured considerably, while foundational studies from the preceding decade are incorporated where they remain methodologically indispensable. The intent is to assist both new investigators in selecting appropriate methods and experienced researchers in calibrating their interpretive frameworks against contemporary standards.

Several recent expert-panel statements have shaped the standards we adopt. The Sies and colleagues consensus on defining roles of specific ROS in cell biology and physiology [2], the Sies–Jones reframing of redox biology around eustress and pleiotropic signalling [23], the Forman–Zhang assessment of antioxidant pharmacology [24], the Kalyanaraman et al. critique of fluorescent probes [20], the AHA consensus statement on ROS measurement [22], and the Murphy et al. Nature Metabolism guidelines [25] together define what a rigorous redox-biology paper now looks like. The contribution of the present review is distinct from any of these and from preceding methodological reviews, including the lead author’s 2015 overview [4]. We do not restate the cellular chemistry of ROS that the consensus statements have already established; nor do we duplicate the philosophical reframing of redox biology articulated by Sies and Jones. Our specific contribution to the 2026 literature is fourfold. First, we integrate, in a single critical synthesis, the parallel maturation of five measurement platforms whose primary studies are typically reviewed in isolation, and we evaluate them against a unified rubric (selectivity, sensitivity, temporal and spatial resolution, quantitative capability, cost, and required expertise). Second, we provide explicit, practice-oriented decision criteria for choosing between methods that are increasingly substitutable on paper but differ in important biochemical interpretation—most consequentially, between HyPer7 and roGFP2-Orp1/Tsa2ΔCR (Section 5.3). Third, we extend the field-level assessment beyond demonstration-level toolboxes into the practical domains of mammalian in vivo translation, biofouling-resistant nanosensor implants, and redox proximity proteomics—all of which have matured between 2020 and 2026 and none of which is adequately covered by earlier reviews. Fourth, and most importantly, we propose that the standards of reporting that are emerging from these advances should now be enforced at the level of editorial policy: the central editorial argument of this review, developed in Section 4 and Section 8, Section 9 and Section 10, is that uncorroborated evidence from a single non-selective probe is best regarded as preliminary rather than as definitive support for a primary mechanistic conclusion in a peer-reviewed publication, and that orthogonal validation is most usefully treated as a strongly recommended baseline rather than as an optional enhancement. In this respect the present article is intended not only as a survey but as an expert-panel position paper that defines the methodological standards we believe the next decade of redox biology should adopt.

## 2. Sources and Cellular Chemistry of Reactive Oxygen Species

**Figure 1:**
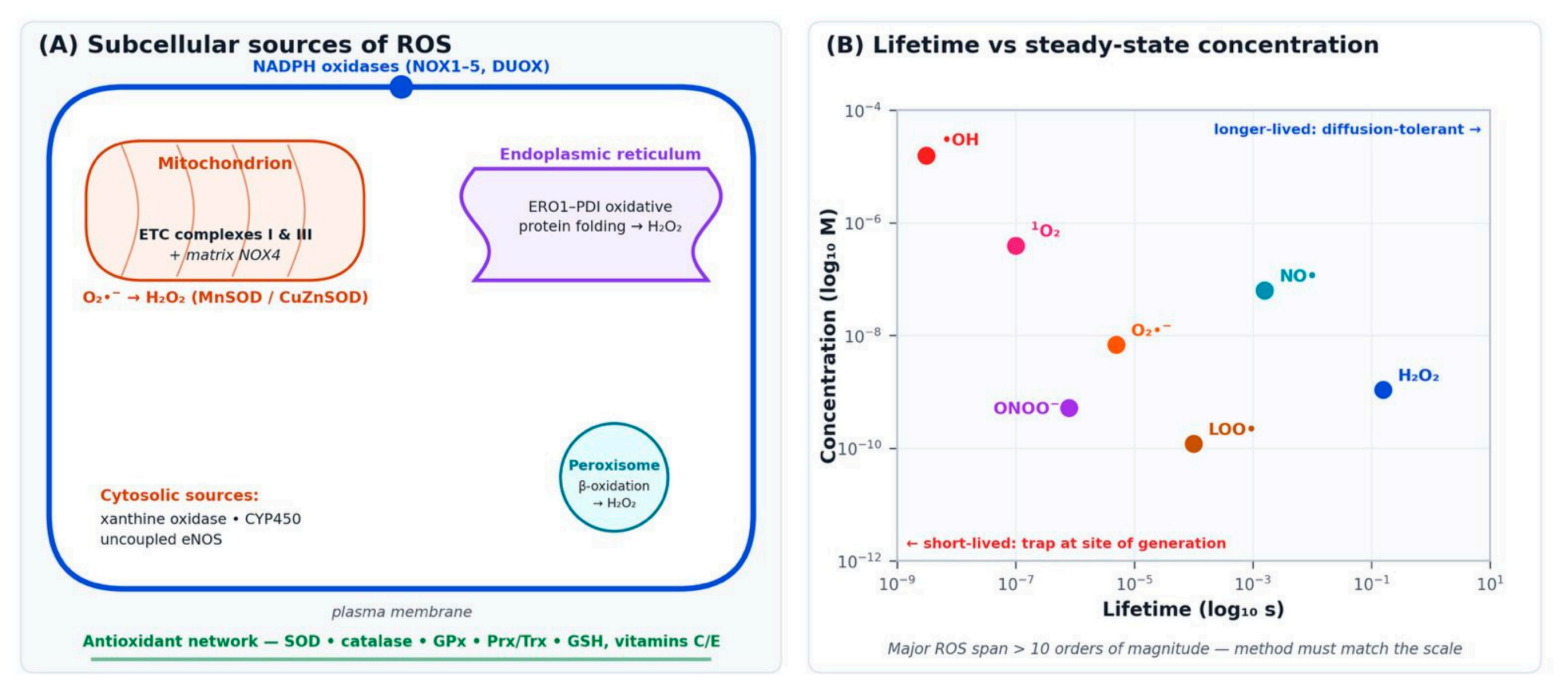
Cellular sources of reactive oxygen species and the species’ lifetimes and concentrations. (**A**) Subcellular sources: mitochondrial electron transport (complexes I and III + matrix NOX4), NADPH oxidases at the plasma membrane, ER (ERO1–PDI oxidative folding), peroxisomal β-oxidation, and cytosolic enzymatic sources (xanthine oxidase, P450, uncoupled eNOS), counterbalanced by the enzymatic and non-enzymatic antioxidant network. (**B**) Lifetimes (x-axis, log seconds) and steady-state concentrations (y-axis, log molar) of major ROSs span more than ten orders of magnitude. Method selection must match this scale: short-lived radicals require trapping at the site of generation, whereas longer-lived oxidants permit diffusion-tolerant chemistry.

A measurement strategy must be matched to the biology of the species being measured. Different ROS have lifetimes spanning thirteen orders of magnitude, react with distinct biomolecular targets, and are generated by distinct cellular machinery (Figure 1). A brief overview of the chemistry frames the methodological considerations that follow.

### 2.1. Endogenous Generation

In aerobic eukaryotic cells, ROS are generated continuously as products of oxygen metabolism. The mitochondrial electron transport chain is the principal cellular source: electron leakage at complexes I and III produces O_2_^•−^, which is dismutated to H_2_O_2_ by MnSOD (matrix) and CuZnSOD (intermembrane space) [26,27]. Mitochondrial ROS production is now recognized as a regulated process modulated by membrane potential, substrate supply, and uncoupling-protein activity, with reverse electron transport at complex I generating substantial H_2_O_2_ bursts during ischemia–reperfusion [27,28,29]. The NADPH oxidase (NOX) family—NOX1–NOX5, DUOX1, DUOX2—generates ROS as its primary enzymatic product rather than as a metabolic side reaction, with tissue distribution and substrate specificity that distinguish dedicated H_2_O_2_ producers (notably NOX4) from O_2_^•−^ generators [30,31,32,33]. Additional enzymatic sources—xanthine oxidase, cytochrome P450, uncoupled eNOS, peroxisomal β-oxidation, and ERO1–PDI-driven oxidative protein folding—collectively contribute to the cellular ROS burden in tissue- and condition-specific ways [3,34,35,36]. The methodologically relevant point is that each source generates a characteristic species (O_2_^•−^ vs. H_2_O_2_) in a defined subcellular compartment, and any measurement strategy must match the species and compartment of interest.

### 2.2. Antioxidant Defenses and the Redox Set Point

The cellular redox set point is set by the coordinated activity of enzymatic and non-enzymatic antioxidants. Enzymatic systems include the SOD isoforms, catalases, glutathione peroxidases, and the abundant peroxiredoxins (up to 1% of total cellular protein), which act both as antioxidants and as redox relays through the thioredoxin and glutaredoxin systems, with NADPH from the pentose phosphate pathway as the ultimate electron source [37,38,39,40]. Non-enzymatic antioxidants—glutathione (1–10 mM), ascorbate, α-tocopherol, urate, and bilirubin—provide complementary radical scavenging [3,34]. Inducible upregulation of cytoprotective genes is coordinated by the Keap1–Nrf2 axis through redox-sensitive cysteines on Keap1 [41,42,43]. For measurement purposes, the practical implications are that (i) any probe entering the cell competes with this dense antioxidant network for its target species, and (ii) the steady-state concentration that a probe reports reflects flux through, rather than absolute generation of, the species of interest.

### 2.3. Implications for Measurement

Several features of ROS chemistry constrain the choice of measurement strategy. First, lifetimes vary enormously: ^•^OH has a half-life of approximately 10^−9^ s and reacts within nanometers of its generation site, while H_2_O_2_ has an effective half-life of seconds to minutes and can diffuse across membranes through aquaporins (peroxiporins). Methods for ^•^OH must therefore use trapping reactions with rate constants near the diffusion limit, while methods for H_2_O_2_ can rely on slower, more selective chemistry. Second, intracellular concentrations span six orders of magnitude—physiological H_2_O_2_ concentrations of 1–10 nM contrast with phagosomal HOCl concentrations of millimolar levels—placing demands on probe sensitivity and dynamic range. Third, compartmentalization is critical: meaningful biological information requires resolution of mitochondrial, cytosolic, nuclear, and ER signals separately, since these compartments maintain distinct redox set points [5,16,44]. Fourth, the act of measurement perturbs the system: probes that consume ROS or alter the redox network can bias the very phenomenon they are intended to observe. Each of the methodological platforms reviewed below addresses these constraints differently, and selection should be guided by the species, compartment, time scale, and quantitative requirement of the experiment.

## 3. Electron Paramagnetic Resonance Spectroscopy

Electron paramagnetic resonance (EPR), also known as electron spin resonance, is the only technique that detects unpaired electrons directly and therefore constitutes the gold standard for the unambiguous identification of free radical species [45,46]. The principle is conceptually identical to nuclear magnetic resonance but exploits electron rather than nuclear spin, with paramagnetic species absorbing microwave radiation at characteristic field strengths. The hyperfine splitting of the resulting spectrum encodes information about the chemical environment of the unpaired electron, allowing identification of the radical species and, in many cases, of its precursor.

### 3.1. Spin Trapping with Nitrones

Because primary radicals such as O_2_^•−^ and ^•^OH have lifetimes too short for direct detection at physiological temperatures, the practical implementation of EPR in biological systems requires spin traps—diamagnetic compounds that react with short-lived radicals to form persistent nitroxide adducts whose EPR spectra are diagnostic of the original radical species. The classical nitrone traps include 5,5-dimethyl-1-pyrroline-N-oxide (DMPO), 5-(diethoxyphosphoryl)-5-methyl-1-pyrroline-N-oxide (DEPMPO), and 5-tert-butoxycarbonyl-5-methyl-1-pyrroline-N-oxide (BMPO) [45,47]. Each yields distinct adducts for O_2_^•−^ and ^•^OH, allowing the two species to be discriminated and quantified in a single sample. DMPO is the most widely used because of its low cost, but its superoxide adduct (DMPO-OOH) decomposes to the same spectrum as the hydroxyl adduct (DMPO-OH), creating an interpretive ambiguity. DEPMPO and BMPO yield more stable superoxide adducts with characteristic phosphorus and carbon hyperfine patterns and are preferred when discrimination is required.

Nitrone spin traps have been validated extensively in chemical, enzymatic, and cellular systems, including isolated mitochondria, perfused hearts, and erythrocytes [45,46]. They have been used to demonstrate the production of O_2_^•−^ by complex I and III of the mitochondrial electron transport chain, by NOX enzymes, and by xanthine oxidase. Practical considerations include the need for relatively high probe concentrations (typically 10–50 mM), the slow rate constants of nitrones with O_2_^•−^ (10^1^–10^2^ M^−1^ s^−1^), and the susceptibility of nitroxide adducts to one-electron reduction by intracellular ascorbate and thiols, which produces systematic underestimation of radical fluxes [45].

### 3.2. Cyclic Hydroxylamine Spin Probes

A more recent development, cyclic hydroxylamine spin probes such as 1-hydroxy-3-methoxycarbonyl-2,2,5,5-tetramethylpyrrolidine (CMH), 1-hydroxy-3-carboxy-2,2,5,5-tetramethylpyrrolidine (CPH), and the mitochondrially targeted Mito-CP, react more rapidly with O_2_^•−^ than nitrones do (rate constants of 10^3^–10^4^ M^−1^ s^−1^) and yield directly stable nitroxides without an intermediate adduct stage [47,48]. These probes are membrane-permeable, can be loaded at lower concentrations (≤1 mM), and are suitable for ex vivo blood, tissue homogenates, isolated organelles, and intact cells. The mitochondrial targeting of Mito-CP through triphenylphosphonium conjugation has been particularly valuable in studies of cardiac ischemia–reperfusion and metabolic disease [48]. A key practical advantage is that quantitative O_2_^•−^ production rates can be calculated from the nitroxide signal kinetics, provided that controls for one-electron reduction are included.

### 3.3. In Vivo and Imaging Applications

Low-field (L-band, ~1 GHz) and very-low-field EPR instrumentation has enabled measurement in small animal models. In vivo EPR oximetry using lithium phthalocyanine or carbon-based probes has provided quantitative oxygen mapping in tumors and ischemic tissues [49]. Spatial EPR imaging at millimeter resolution has been demonstrated for skin and superficial organs, although deeper tissues remain inaccessible. Recent rapid-scan EPR techniques, in which the magnetic field is scanned through resonance faster than the spin relaxation time, have improved sensitivity for short-lived radicals and have begun to find application in cellular preparations [46].

### 3.4. Limitations

The principal practical limitations of EPR are: (i) instrumentation cost (typically ≥ €200,000 for a research-grade X-band spectrometer), restricting the technique to specialist laboratories; (ii) modest sensitivity (typical detection limits of 10^−7^–10^−9^ M nitroxide), often below endogenous radical fluxes in healthy cells; (iii) the requirement for relatively high probe concentrations, which can perturb the system being measured; and (iv) the inability to resolve subcellular compartments without targeted probes. Despite these limitations, EPR remains indispensable when unambiguous identification of a specific radical species is required—for instance, in mechanistic studies of drug-induced oxidative damage, in validating the selectivity of fluorescent probes, or in identifying the radical precursor of an observed cellular response [22,46].

## 4. Small-Molecule Fluorescent Probes

Fluorescent probes are the most widely used tools for ROS measurement in living systems because of their compatibility with standard microscopy, flow cytometry, and microplate readers. The field has matured substantially over the past decade, with the recognition that early non-specific probes such as DCFH-DA must be replaced—or at least supplemented—by chemoselective alternatives whenever quantitative or species-specific claims are made [9,20,22].

### 4.1. The Limitations of DCFH-DA

2′,7′-Dichlorofluorescin diacetate (DCFH-DA) crosses the cell membrane passively, is hydrolyzed by intracellular esterases to the trapped polar form DCFH, and is then oxidized to fluorescent 2′,7′-dichlorofluorescein (DCF). Although widely cited as an “indicator of intracellular hydrogen peroxide,” DCFH is in fact oxidized only slowly by H_2_O_2_ alone (negligibly under physiological conditions); the principal oxidants are peroxynitrite, hydroxyl radical, hypochlorite, and various heme proteins [9,20,50]. DCFH also undergoes light-driven autoxidation that confounds interpretation, and the oxidation product DCF is itself redox-active and can sensitize further oxidation, leading to artificial signal amplification. DCFH-DA cannot be used to quantify H_2_O_2_ or to compare oxidative loads across experiments unless rigorous controls (catalase quenching, parallel measurement with selective probes) are included [22]. Despite these well-documented limitations, DCFH-DA continues to dominate the ROS literature, and a substantial fraction of contemporary papers report only “increased DCF fluorescence” as evidence of oxidative stress—a claim that contemporary peer review should no longer accept without corroboration.

This critique should not be read as a blanket prohibition. DCFH-DA retains a defensible role as a low-cost, broadly accessible reporter of bulk cellular oxidant load in two well-defined contexts: (i) preliminary screening assays—for example, in early-stage compound libraries, environmental toxicology pilots, or resource-limited settings where instrument access to mass spectrometry, EPR, or genetically encoded biosensors is not feasible—and (ii) qualitative confirmation of an expected oxidative perturbation in parallel with a chemoselective probe, where DCFH-DA serves as an internal sanity check rather than as the primary readout [23,24,51]. Three control conditions are mandatory whenever the probe is used: a catalase- or N-acetylcysteine-quenchable signal to demonstrate redox dependence; a parallel measurement with a chemoselective probe (boronate-based for H_2_O_2_, DHE/HPLC for O_2_^•−^) on at least a representative subset of conditions; and explicit reporting of probe loading, illumination conditions, and incubation times to permit assessment of autoxidation artifacts. With these provisos, DCFH-DA can support hypothesis-generating workflows; without them, its results should not be presented as evidence of any specific reactive species. The distinction we wish to maintain is between rejecting DCFH-DA as a tool (we do not) and rejecting the interpretation of unguarded DCF fluorescence as a quantitative measure of H_2_O_2_ (we do).

We frame this not as a rigid editorial rule, but as a strong methodological recommendation. Given the magnitude of the autoxidation, light-driven artefact, and species-cross-reactivity problems documented over more than a decade [9,20,21,22], any manuscript whose principal mechanistic claim about a specific reactive species rests exclusively on DCFH-DA fluorescence—without orthogonal validation by EPR, by a chemoselective fluorescent probe with a distinct chemistry, by LC-MS/MS quantification of a defined oxidation product, or by a genetically encoded biosensor—should, in our view, be interpreted with caution and ideally corroborated before it is relied upon to support a primary mechanistic conclusion in journals that intend to set the methodological standard for the field. This is not a counsel of perfection; it reflects the level of evidence we believe is needed to defend a quantitative or species-specific claim with confidence. Therefore, as a best practice in modern redox biology, researchers are encouraged to complement DCFH-DA measurements with orthogonal techniques when drawing specific mechanistic conclusions, thereby providing a more rigorous evidence base. The cost of orthogonal validation has fallen substantially over the past decade as commercial probes, biosensor reagents, and core-facility EPR and LC-MS/MS access have become widely available; the methodological gap is now one of culture and editorial policy, not of resource access.

### 4.2. Boronate-Based Probes for Hydrogen Peroxide

The most important advance in small-molecule H_2_O_2_ detection has been the development of activity-based probes incorporating an aryl boronate or boronic acid pinacol ester group as the reactive trigger [8,10,52]. Boronates undergo a chemoselective reaction with H_2_O_2_ (and, more rapidly, with peroxynitrite) yielding the corresponding phenol with concomitant unmasking of fluorescence. The Chang laboratory introduced the prototype Peroxyfluor-1 (PF1) and subsequently developed an extensive family including Peroxy Green-1 (PG1), Peroxy Crimson-1 (PC1), Peroxyresorufin-1 (PR1), and the mitochondrially targeted MitoPY1 [8,9,53]. MitoPY1 incorporates a triphenylphosphonium cation that drives accumulation in the mitochondrial matrix in proportion to the membrane potential, enabling selective imaging of mitochondrial H_2_O_2_ generation in mammalian cells, *C. elegans*, zebrafish, and mice [53,54].

Boronate probes provide approximately 1000-fold selectivity for H_2_O_2_ over O_2_^•−^, NO, and most other ROS, although peroxynitrite reacts approximately 10^6^-fold faster than H_2_O_2_ and must be considered when ONOO^−^ is plausibly present [10,52]. Newer-generation probes incorporate near-infrared emission for in vivo imaging (Peroxy Caged Luciferin-1, PCL-1; IR-FLAP), photoacoustic readout, and ratiometric reporting that enables semi-quantitative interpretation [25,53,55]. The boronate platform now extends to bioluminescent and chemiluminescent readouts and to so-called “trapping probes” that label the nucleophile produced after oxidation, enabling mass-spectrometry-based identification of redox-modified targets [10]. A comprehensive review of the chemistry, kinetics, and biological applications of boronate probes through 2022 is available [9].

### 4.3. Probes for Superoxide and Other ROS

Dihydroethidium (hydroethidine, DHE) and its mitochondrially targeted analog MitoSOX Red are widely used for O_2_^•−^ detection. The reaction with O_2_^•−^ yields the diagnostic product 2-hydroxyethidium (2-OH-E^+^), which differs in fluorescence excitation spectrum from the non-specific oxidation product ethidium [56]. Reliable interpretation requires HPLC separation of the two products, since fluorescence microscopy alone cannot distinguish them; flow cytometry signals reported as “DHE fluorescence” without HPLC validation should be interpreted with caution [22,56,57]. Despite these caveats, the DHE/MitoSOX system, when used with proper analytical separation, remains the most accessible approach for cellular O_2_^•−^ measurement and has been extensively cross-validated against EPR.

A second generation of superoxide-selective tools has emerged that addresses the principal limitations of the DHE/MitoSOX family—incomplete chemoselectivity, the requirement for HPLC product separation, and weak fluorescence yield at physiological O_2_^•−^ fluxes. Triphenylphosphonium-conjugated hydroethidine analogs—most prominently Mito-HE (also termed mito-DHE) and the more recent o-Mito-Phenyl-B(OH)_2_ derivatives—drive accumulation in the mitochondrial matrix in a membrane-potential-dependent manner and yield diagnostic 2-hydroxy products that, when quantified by LC-MS/MS, provide absolute matrix O_2_^•−^ generation rates in isolated mitochondria, perfused organs, and cultured cells [55,58,59,60]. Quantitative correction for the membrane-potential-dependent uptake fraction is essential whenever mitochondrial bioenergetics are perturbed, since changes in ΔΨ_m_ alter probe accumulation independently of radical flux. Chemiluminescent O_2_^•−^ probes—including coelenterazine analogs, MCLA (methyl-Cypridina-luciferin analog), and L-012—provide background-free detection in cellular and tissue suspensions because no excitation light is required, eliminating photo-induced artifacts that confound fluorescent probes in extended time-course experiments. Coelenterazine derivatives have been used in luciferase-coupled formats for in vivo bioluminescence imaging of NADPH-oxidase-derived O_2_^•−^ in murine inflammation models, providing a non-invasive readout that complements the more invasive EPR or LC-MS/MS endpoints. None of these tools is free of interferences (peroxidase-mediated artifacts for L-012, alternative oxidants for coelenterazine), and orthogonal validation against EPR or 2-hydroxy-product LC-MS/MS remains the gold standard for mechanistic claims. Nevertheless, the combination of TPP-conjugated hydroethidine analogs with LC-MS/MS quantification has emerged as the practical reference method for absolute mitochondrial O_2_^•−^ quantification in cellular and small-animal preparations [58,60].

Amplex Red (10-acetyl-3,7-dihydroxyphenoxazine), a non-fluorescent substrate that is oxidized to fluorescent resorufin in a horseradish-peroxidase-catalyzed reaction with H_2_O_2_, provides a sensitive (sub-nanomolar) and quantitative assay for extracellular H_2_O_2_ release [61]. The probe cannot enter cells and the peroxidase cannot cross membranes, so the assay reports specifically on H_2_O_2_ that has effluxed to the extracellular space—a useful boundary condition for measuring NOX activity in isolated membranes or H_2_O_2_ efflux through peroxiporins. For singlet oxygen, the trans-1-(2′-methoxyvinyl)pyrene (t-MVP) probe and Singlet Oxygen Sensor Green (SOSG) are the standard fluorogenic options [62]. Hypochlorous acid is detected with aminophenyl-fluorescein (APF) and 3′-(p-aminophenyl) fluorescein, which react selectively with HOCl over other ROS [63].

### 4.4. Subcellular Targeting

A defining advance of the past decade has been the development of probes targeted to specific subcellular compartments. Mitochondrial targeting is achieved through triphenylphosphonium (TPP^+^) conjugation, exploiting the negative-inside potential of the inner membrane [64]. ER targeting uses tunicamycin- or methylsulfonamide-based localization signals; lysosomal targeting employs morpholine- or amine-based weak bases that protonate at acidic pH [65]. Nuclear targeting is more challenging but has been achieved with DNA-binding moieties such as Hoechst-derived units. Probes have been published for simultaneous dual-organelle imaging—for example, mitochondrial and ER H_2_O_2_ during apoptosis [65]—enabling investigation of inter-organelle redox communication previously inaccessible to small-molecule chemistry.

### 4.5. Practical Considerations and Validation

Several practical guidelines now apply to the responsible use of fluorescent probes: (i) every probe should be validated in the experimental system through positive controls (exogenous H_2_O_2_ or generators) and quench controls (catalase for H_2_O_2_, SOD or its mimetics for O_2_^•−^); (ii) photo- and dark-stability should be characterized for each cell type and exposure regimen; (iii) loading conditions should be optimized to avoid probe sequestration in lysosomes or autoxidation in serum-containing medium; (iv) where possible, ratiometric probes should be preferred over intensity-based reporters because they correct for variations in loading, illumination, and cell thickness [11,20,22]. Recent expert consensus statements provide detailed practical protocols [3,9,22].

### 4.6. Lipid-Peroxidation Probes and Ferroptosis Detection

The rapid growth of ferroptosis research over precisely the period covered by this review has made lipid-peroxidation detection a methodological priority in its own right, deserving more than the passing mention it has traditionally received in general ROS surveys. Ferroptosis is an iron-dependent, non-apoptotic form of regulated cell death driven by the accumulation of phospholipid hydroperoxides [66], whose central enzymatic brake is the selenoprotein glutathione peroxidase 4 (GPX4); genetic or pharmacological inactivation of GPX4 is sufficient to trigger lethal lipid peroxidation in mammalian cells and in vivo [67,68]. Because the proximal species is a lipid hydroperoxide rather than a freely diffusible small oxidant such as H_2_O_2_, its detection requires dedicated tools.

Two fluorescent probes dominate practical use. C11-BODIPY 581/591 is a ratiometric, membrane-incorporating probe whose emission shifts from red to green upon oxidation of its polyunsaturated tail, allowing lipid peroxidation to be quantified by microscopy or flow cytometry with an internal ratiometric correction for probe loading [69,70]. Liperfluo, a spirohydroxylated perylene derivative developed from an earlier swallow-tailed perylene probe, is a lipid-hydroperoxide-selective reporter with high sensitivity and improved solubility, widely used to follow lipid-peroxidation flux in ferroptosis assays [71,72]. Both share the generic limitations of small-molecule probes discussed in Section 4.5—an essentially irreversible response, potential perturbation of membrane order, and the need for careful loading controls—and neither identifies the specific oxidized lipid species, which still requires the LC-MS/MS oxidative-lipidomics approaches discussed in Section 6.

In practice, robust ferroptosis detection therefore mirrors the orthogonal-validation logic advocated throughout this review: a ratiometric or hydroperoxide-selective fluorescent readout (C11-BODIPY 581/591 or Liperfluo) is most convincing when corroborated by a functional manipulation of the GPX4 axis—for example, rescue by lipophilic radical-trapping antioxidants such as ferrostatin-1 or liproxstatin-1, or by iron chelation—and, where feasible, by mass-spectrometric identification of the oxidized phospholipids. Readers working in cancer or neurodegeneration, where ferroptosis is now a major focus, are referred to dedicated reviews of ferroptosis mechanisms and detection for comprehensive practical guidance [73].

## 5. Genetically Encoded Fluorescent Biosensors

Genetically encoded biosensors represent the most significant methodological advance in redox biology of the last fifteen years. Unlike small-molecule probes, which are loaded transiently and may distribute non-uniformly within cells, fluorescent protein-based sensors are expressed from DNA constructs, can be targeted with precision to any subcellular compartment by appropriate signal sequences, and permit longitudinal observation in living cells, organoids, and intact organisms [11,74]. Two principal families dominate the field: HyPer sensors based on circularly permuted yellow fluorescent protein fused to bacterial OxyR, and roGFP variants harboring engineered surface cysteines that report redox potential ratiometrically.

### 5.1. The HyPer Family for Hydrogen Peroxide

The HyPer biosensor, introduced by Belousov and colleagues in 2006 and subsequently refined into HyPer-2, HyPer-3, HyPer-Red, and the high-performance HyPer7, is a circular permutation of yellow fluorescent protein (cpYFP) inserted into the regulatory domain of the bacterial peroxide-sensing protein OxyR [75,76]. Reaction of OxyR cysteines with H_2_O_2_ generates an intramolecular disulfide bond that alters the conformation of the chromophore, producing a ratiometric change in the excitation spectrum (peaks shift between 420 nm and 500 nm) read out at the constant 516 nm emission. This ratiometric design corrects for variations in expression level, illumination intensity, and cell thickness—providing genuinely quantitative H_2_O_2_ measurements [13,74].

HyPer7, the most recent generation, increases dynamic range tenfold over HyPer3 by incorporating the OxyR domain from Neisseria meningitidis rather than Escherichia coli and provides reliable signals at physiological H_2_O_2_ concentrations (low nanomolar) where earlier variants were insensitive [74]. HyPer7 has demonstrated compartment-specific H_2_O_2_ fluxes in the mitochondrial matrix versus the cytosol, tracked redox dynamics during T-cell activation and macrophage polarization, and monitored regenerating zebrafish tissue at single-cell resolution [74]. HyPer-Red, with red-shifted spectra, allows multiplexing with green-fluorescent reporters of metabolism or signaling [13,75].

A persistent limitation of all HyPer variants is residual pH sensitivity arising from the protonation state of the chromophore; the SypHer construct, which retains pH sensitivity but loses redox responsiveness, is therefore co-expressed in many studies as an internal pH control [75,76]. Recent variants (HyPerRed, pHRed-mt) have reduced pH dependency, but careful pH control or correction remains essential for quantitative interpretation. HyPer sensors do not respond to glutathione redox potential, peroxynitrite, or other ROS, providing genuinely H_2_O_2_-specific measurements when properly applied.

### 5.2. roGFP-Based Sensors and Redox Relays

Reduction-oxidation-sensitive GFP (roGFP) variants, developed by Remington and colleagues, contain engineered surface cysteines (typically Cys147 and Cys204) whose disulfide-bond formation alters chromophore protonation and produces ratiometric excitation changes [11,77]. The two principal variants, roGFP1 and roGFP2, differ in pKa and dynamic range. Because thiol-disulfide kinetics with H_2_O_2_ alone are slow at physiological concentrations, roGFP is most useful when fused with a redoxin partner that provides catalytic specificity and rate enhancement [11,77].

roGFP2-Orp1 (and the higher-sensitivity roGFP2-Tsa2ΔCR) couples roGFP2 to the yeast peroxiredoxin Orp1 or Tsa2, which reacts with H_2_O_2_ approximately 10^5^-fold faster than free thiols and transfers the oxidative equivalent to roGFP2 through a disulfide-exchange relay [11,78]. The result is a sensor that responds rapidly and selectively to H_2_O_2_ over a wide concentration range and is largely insensitive to glutathione redox potential. The complementary Grx1-roGFP2 fusion couples roGFP2 to glutaredoxin-1 and reports the glutathione redox potential (E_GSH_) in the millivolt range, providing a quantitative readout of one of the most important cellular redox couples [11,74,77,79,80,81]. Both fusion sensors have been targeted to the mitochondrial matrix, intermembrane space, cytosol, nucleus, ER lumen, peroxisomes, and the chloroplast stroma in plant systems.

A critical advantage of the roGFP-Orp1 platform over HyPer variants is complete pH independence—the sensor reports disulfide bond formation rather than chromophore protonation, and its ratiometric signal is therefore unaffected by the substantial pH changes that accompany mitochondrial depolarization, nutrient withdrawal, or acidic organelles [78,82]. This makes roGFP2-Orp1 the sensor of choice in experimental contexts where pH cannot be controlled. Its dynamic range (OxD 0 to 1, corresponding to approximately 1–200 nM steady-state H_2_O_2_) has been validated using kinetic modelling against the known peroxidase rate constants and confirmed by parallel LC-MS/MS measurement of probe oxidation products [78,82]. In *C. elegans* and zebrafish, genetically encoded roGFP2-Orp1 and Grx1-roGFP2 sensors expressed as stable transgenics have transformed in vivo redox biology by enabling single-cell resolution measurements during development, ageing, and pathological stress without any exogenous probe loading [18,83].

### 5.3. HyPer7 Versus roGFP2-Orp1/Tsa2ΔCR: Choosing Between H_2_O_2_ Biosensors

**Figure 2:**
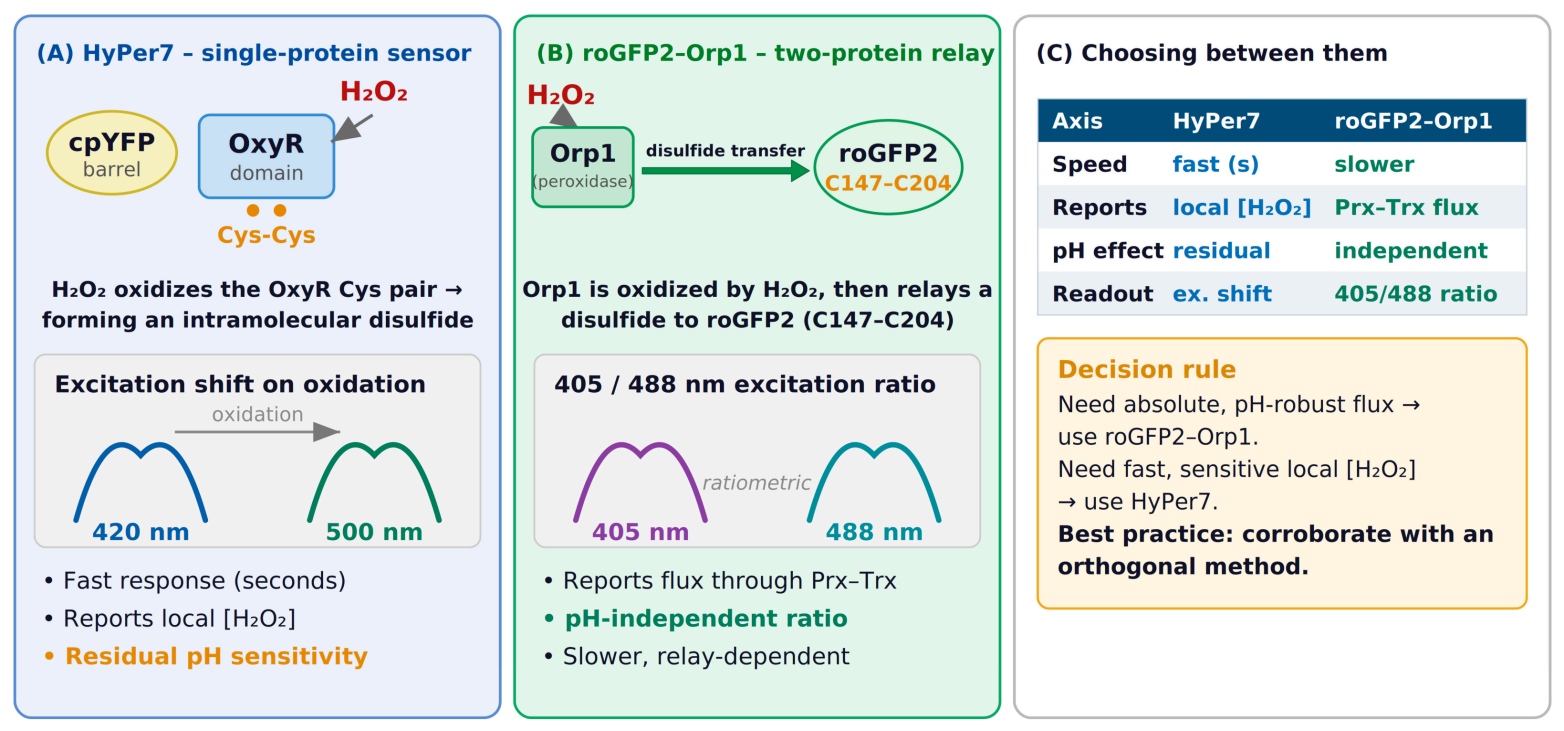
Mechanistic and decision-rule comparison of HyPer7 and roGFP2-Orp1/Tsa2ΔCR. (**A**) HyPer7 senses H_2_O_2_ through an intramolecular OxyR disulfide-bond rearrangement that shifts the cpYFP excitation peak from 420 to 500 nm; reaction is fast (seconds) and reports local [H_2_O_2_], with residual pH sensitivity. (**B**) roGFP2-Orp1 uses an intermolecular two-protein relay: Orp1 is oxidized by H_2_O_2_ and transfers a disulfide to roGFP2 (C147–C204), shifting the 405/488 ratio; the sensor reports flux through the Prx–Trx pathway and is pH-independent. (**C**) Comparison axes and the decision rule emerging from these mechanistic differences. Use HyPer7 when low-nanomolar sensitivity, fast kinetics, and pH-controlled cytosol are required; use roGFP2-Orp1 for variable-pH compartments or to read reductive capacity; use both in parallel when discordance is biologically informative.

Investigators frequently must choose between HyPer7 and the roGFP2-Orp1/roGFP2-Tsa2ΔCR family for a given experiment, and the choice is non-trivial because both sensors are ratiometric, both are genetically encoded, both can be targeted to any subcellular compartment, and both report H_2_O_2_ with high specificity. The boundary conditions that should drive the selection cluster along four axes: pH sensitivity, dynamic range and detection limit, response kinetics, and the underlying biochemical quantity reported (Figure 2).

First, pH sensitivity. HyPer7 retains residual pH sensitivity inherited from the cpYFP scaffold, and although co-expression of SypHer or pHRed-mt as an internal pH control is now standard, the correction assumes that the pH sensor and the HyPer sensor experience identical microenvironments—an assumption that is difficult to verify rigorously, particularly in subcellular compartments with steep pH gradients. In experimental settings where matrix or organelle pH is expected to change substantially—mitochondrial depolarization during ischemia–reperfusion, uncoupler treatment, lysosomal-axis investigation, or nutrient-withdrawal-induced autophagy—even a well-corrected HyPer7 signal carries non-trivial residual artefact. Under these conditions roGFP2-Orp1/Tsa2ΔCR is categorically superior because the ratiometric signal arises from disulfide-bond formation rather than chromophore protonation and is intrinsically pH-independent [78,82]. Conversely, in cytosolic experiments where pH is well buffered (mammalian cytosol typically holds pH 7.0–7.4 within a tenth of a unit under physiological conditions), the HyPer7 pH penalty is small, and its other advantages dominate.

Second, dynamic range and detection limit. HyPer7 reaches reliable signal change at low-nanomolar H_2_O_2_ concentrations (close to the physiological 1–10 nM range) where earlier HyPer variants and unmodified roGFP2 were uninformative; this makes HyPer7 the better choice for resolving steady-state physiological fluxes and small adaptive perturbations [74]. roGFP2-Orp1 has a usable dynamic range of approximately 1–200 nM steady-state H_2_O_2_ [78,82], which encompasses adaptive and pathological states but underperforms HyPer7 at the lowest end of the physiological range. The roGFP2-Tsa2ΔCR variant, in which the resolving cysteine of yeast peroxiredoxin Tsa2 is mutated to extend the disulfide-relay half-life, partially closes this sensitivity gap and is the preferred roGFP-relay variant when low-nanomolar resolution is needed under conditions of pronounced pH change.

Third, response kinetics. HyPer7 undergoes an intramolecular OxyR conformational change after H_2_O_2_ reaction with single-protein kinetics on the order of seconds. The roGFP2-Orp1 platform requires an intermolecular disulfide exchange between Orp1 and roGFP2, the rate of which depends on local Orp1 concentration and on competing reductive pathways; effective response times are typically tens of seconds and are sensor-concentration dependent. For experiments resolving rapid, oscillatory, or pulsed H_2_O_2_ dynamics—for example, calcium-coupled mitochondrial bursts, sub-second NOX activation, or signalling-relevant pulses during T-cell activation—HyPer7 has the kinetic advantage. roGFP-relay sensors are appropriate for steady-state and slowly evolving fluxes (minutes to hours).

Fourth, and most subtly, the two sensor families do not report the same biochemical quantity. HyPer7 reports the local H_2_O_2_ concentration through the kinetics of OxyR cysteine oxidation, with the steady-state signal reflecting the balance of influx and the OxyR reduction reaction. roGFP2-Orp1, in contrast, reports flux through the peroxiredoxin–thioredoxin reductive pathway: the steady-state ratio reflects the kinetic balance between Orp1 oxidation by H_2_O_2_ and reduction in Orp1–roGFP2 disulfides by the cellular thioredoxin system. In systems where thioredoxin or thioredoxin-reductase activity is altered—pharmacological inhibition of TrxR (auranofin, PMX464), genetic knockdown, oxidative inactivation of TrxR, or compartments with limited NADPH supply—the two sensors will report divergent values, and the divergence is biologically informative rather than a technical failure. HyPer7 will continue to track local H_2_O_2_, while roGFP2-Orp1 will record the integrated effect of H_2_O_2_ production and reductive throughput. In cancer pharmacology, where TrxR inhibitors are an active class of investigational agents, this distinction is biologically important: dual-sensor readouts can in principle separate a primary increase in H_2_O_2_ generation from a secondary collapse of reductive capacity.

A practical decision rule emerges. Use HyPer7 when the experiment requires the highest sensitivity at low-nanomolar H_2_O_2_, when fast kinetics matter, and when pH is well controlled or pH-correction is feasible. Use roGFP2-Orp1 (or roGFP2-Tsa2ΔCR) when pH is uncontrolled or expected to vary, when the biological question concerns the integrated reductive capacity of the cell, or when long-term comparative measurements across heterogeneous cellular states require pH independence. Where feasible, the most defensible experimental design uses both sensors in parallel: concordance increases confidence in the primary H_2_O_2_ readout, while informative discordance enables mechanistic dissection of the redox network rather than merely its endpoint.

### 5.4. Translation to Mammalian In Vivo Models

The maturation of HyPer7 and roGFP2-Orp1 in invertebrate and zebrafish models has been followed, over the past five years, by their first systematic translation to mammalian in vivo systems [84,85,86,87]. Three delivery strategies have emerged. First, recombinant adeno-associated virus (rAAV) vectors carrying biosensor cassettes under tissue-specific promoters allow durable, non-integrating expression in post-mitotic and slowly dividing tissues. Capsid serotypes have been selected empirically for organ tropism—AAV9 for cardiac and skeletal muscle, AAV-PHP.eB and AAV-PHP.B for central nervous system after systemic injection, AAV8 for hepatocytes, AAV6 for skeletal muscle by intramuscular delivery, and AAV-DJ for broad in vitro applications. Second, lentiviral and retroviral vectors are used where stable integration into proliferating cell populations is required, particularly for hematopoietic and immune-cell studies. Third, transgenic and knock-in mouse lines expressing biosensors from the Rosa26 locus or from endogenous redox-relevant loci provide the cleanest expression but at substantial cost in time and breeding effort. The choice among these depends on the biological question: AAV is preferred for adult-onset, organ-restricted, reversible expression; transgenics are preferred for developmental studies and for guarantees of expression in every cell of a lineage [84,85].

Three application areas have demonstrated the value of mammalian biosensor expression. In skeletal muscle, AAV9- or AAV6-delivered cytosolic and mitochondrial-targeted HyPer7 has resolved compartment-specific H_2_O_2_ dynamics during contractile activity, ischemia–reperfusion, and aging-associated sarcopenia in living mice [58,59,88]. Intravital two-photon microscopy through dorsal skinfold or surgically exposed tibialis anterior windows has resolved redox transients at single-fiber and single-mitochondrion resolution. In liver, AAV8-mediated hepatocyte-restricted expression (using the thyroxine-binding globulin or hepatocyte-specific albumin promoter) has been used to study redox dynamics in models of non-alcoholic steatohepatitis, acetaminophen toxicity, and drug-induced liver injury, with biosensor signals correlating with conventional damage markers and providing temporal information unattainable by post-mortem histology. In brain, intracranial or intracerebroventricular injection of AAV-PHP.eB carrying neuronal (Syn1, CamKIIα) or astrocytic (GFAP) promoter-driven biosensors has revealed cell-type-specific redox responses to ischemic stroke, status epilepticus, and neurodegenerative protein aggregation. Cranial-window two-photon imaging of these preparations has resolved subcellular redox events with synaptic resolution in awake, behaving animals.

Several practical caveats apply to this growing literature [84,85,89]. (i) Tissue penetration of two-photon excitation is limited to approximately 500–800 μm even with optimized objectives and clearing protocols, restricting most measurements to surface-accessible structures or to tissue windows that introduce surgical perturbation. (ii) AAV-mediated expression is heterogeneous among cells of an apparently uniform population, requiring per-cell ratiometric normalization rather than population averaging. (iii) Promoter leakiness can produce off-target expression that confounds cell-type-specific conclusions; rigorous immunohistochemical or single-cell sequencing validation of the targeted cell type is essential. (iv) Long-term AAV expression (beyond approximately 6 months) can elicit anti-capsid immune responses that reduce signal in some inbred strains. (v) Calibration in vivo remains the principal unsolved challenge; minimum and maximum-signal references are typically obtained ex vivo on a parallel cohort, with the in vivo trace expressed as fractional oxidation between these bounds. If current technical obstacles—particularly in vivo calibration, delivery homogeneity, and long-term expression stability—can be surmounted, AAV-delivered biosensors may, within the next decade, transition from proof-of-concept demonstrations to a standard component of the mammalian redox physiologist’s toolkit and increasingly inform clinical-precursor biomarker work alongside, rather than in immediate replacement of, the existing fluorescent-probe literature.

Several practical barriers nonetheless temper this translational optimism and warrant explicit acknowledgement. First, delivery remains the rate-limiting step: genetically encoded biosensors must be introduced into the target tissue either by transgenesis—as achieved for HyPer and roGFP2-based reporters in zebrafish and in cardiomyocyte-specific transgenic mice [90,91]—or by viral vectors, most commonly adeno-associated virus (AAV), whose finite packaging capacity, variable tissue tropism, pre-existing immunity, and mosaic transduction efficiency all constrain uniform, quantitative expression across a tissue [92]. Second, long-term expression raises safety and physiological-perturbation concerns: sustained high-level expression of an H_2_O_2_-reactive protein such as HyPer7 or roGFP2-Orp1 can, in principle, buffer or distort the very redox signal it is intended to report, so that constitutive sensor expression must be shown not to alter baseline redox homeostasis or cell viability [75,79]. Third, in vivo quantification is complicated by limited optical access, motion, autofluorescence, and pH or temperature gradients that are difficult to control in living tissue, so that the absolute calibration achievable in cultured cells is rarely attainable in situ. Finally, any eventual clinical or diagnostic translation would face a substantial regulatory burden—encompassing vector biosafety, long-term genomic-integration risk, and immunogenicity—that currently confines these tools to research and pre-clinical use. We therefore regard genetically encoded biosensors as the most informative available approach for compartment-resolved redox imaging in experimental mammalian systems, while cautioning that their routine in vivo and translational deployment remains an aspiration constrained by delivery, safety, and regulatory factors rather than an established reality.

### 5.5. Other Genetically Encoded Sensors

A wider toolkit has emerged in recent years. FRET-based oxyFRET and perFRET sensors for H_2_O_2_ incorporate Cerulean and Venus fluorescent proteins flanking redox-responsive domains; TrxRFP1 reports thioredoxin redox state; OHSer detects hydroxyl radical via reactive serine chemistry; pHaROS and GRX1-pHaROS provide combined pH and redox readouts; and CROST and FROG/B sensors are based on alternative fluorescent protein scaffolds [11]. For nitric oxide, the geNOps family (G-geNOp, B-geNOp, R-geNOp), based on a bacterial NO-binding domain, provides selective NO detection without the cross-reactivity that plagues small-molecule NO indicators [93]. Selection of the appropriate sensor depends on the target species, required dynamic range, desired subcellular compartment, and need for multiplexing with other reporters; a comprehensive comparative evaluation has been published [11].

### 5.6. Practical Considerations

Genetically encoded sensors require introduction of the sensor gene into target cells, which adds experimental complexity (transfection, viral transduction, generation of stable cell lines, or transgenic animals). Expression levels must be optimized: too low yields poor signal, too high can saturate the cellular antioxidant network and perturb the very phenomenon under study. Calibration in living cells remains imperfect and is typically performed by comparing the in situ signal to fully oxidized (DTT/diamide-treated) and fully reduced reference conditions. A specific and underappreciated practical difficulty is that the oxidized/reduced reference points are almost always obtained ex vivo—on a parallel sample, after permeabilization, or under non-physiological exposure to calibrating agents—while the experimental measurement is in vivo. The two regimes differ in fluorescent-protein photophysics (cellular versus buffer environment), in local redox-buffer capacity, and in the effective intracellular concentration of the calibrating agent reaching the sensor, all of which introduce systematic uncertainty in the derived OxD or fractional-oxidation values.

Calibration in mammalian in vivo systems is the field’s central unresolved problem—the “Achilles heel” of contemporary biosensor-based redox biology—and warrants more than passing mention. Three modelling approaches now offer a path that may, within the next several years, supplant or substantially reduce the dependence on ex vivo reference points. The first is the kinetic-model framework developed for roGFP2-Orp1 by Pak and colleagues [78,82], in which the steady-state ratiometric signal is interpreted as a function of (i) the second-order rate constant of Orp1 oxidation by H_2_O_2_, (ii) the rate of disulfide-relay transfer from Orp1 to roGFP2, and (iii) the cellular thioredoxin-mediated reduction rate. Given measured rate constants for the peroxiredoxin and thioredoxin systems, the framework solves for steady-state H_2_O_2_ directly from the observed sensor ratio, sidestepping the in vivo–ex vivo gap entirely. The second is dynamic-flux deconvolution, in which time-resolved sensor traces during a defined perturbation (e.g., bolus injection of an exogenous oxidant or pharmacological scavenger) are fit to a compartmental ODE model whose parameters include both the local generation rate and the antioxidant clearance rate. This approach yields fluxes rather than concentrations and is the natural framework for interpreting sensors that report flux through a redoxin pathway. The third, more recent, is a Bayesian inference framework that propagates uncertainty in rate constants, cellular sensor concentration, and signal-to-noise into credible intervals on the inferred H_2_O_2_ trajectory, replacing point estimates with explicit uncertainty distributions.

All three approaches require that the rate-constant inputs—particularly the cellular Trx/TrxR-mediated reduction rate—be measured rather than assumed. This is itself a non-trivial challenge in mammalian tissues, where Trx pool size, TrxR activity, and NADPH supply vary by cell type, metabolic state, and pathology. The roGFP2-Orp1 framework has been successfully applied to cultured mammalian cells and to zebrafish; extension to mammalian in vivo settings is technically feasible but has not yet been published at scale. An analogous formalism for HyPer7 is in early development and remains a methodological priority for the field; the slower disulfide kinetics and the OxyR-domain reduction by the Trx system make HyPer7 amenable to comparable kinetic modelling, but the parameters have not yet been comprehensively measured. Until model-based calibration is broadly deployed, mammalian in vivo studies should report in vivo signals as fractional oxidation between explicit ex vivo bounds—never as inferred absolute concentrations—and should accompany every absolute concentration claim with cross-validation by an orthogonal method (LC-MS/MS quantification of probe oxidation products is the current reference standard). The emergence of a community-accepted, model-based calibration framework for mammalian in vivo redox biology would be among the most consequential single advances of the next five years and should be a target for collaborative effort across the laboratories developing the major sensor platforms.

Despite these complexities, the targeting precision, ratiometric quantification, and longitudinal capability of genetically encoded sensors have made them the gold standard for compartment-specific redox measurements in cellular and small-organism systems [11,74].

## 6. Mass Spectrometry and Chromatographic Methods

Mass spectrometry (MS) does not measure short-lived ROS directly but provides the gold standard for quantification of stable oxidation products and trapped radical adducts. Three principal applications dominate: (i) MS quantification of fluorescent-probe products for absolute calibration; (ii) measurement of validated biomarkers of oxidative damage (lipid peroxidation, DNA oxidation, protein nitration); and (iii) redox proteomics—proteome-wide identification of cysteine oxidation states [3,94,95].

### 6.1. Quantification of Fluorescent-Probe Products

Liquid chromatography–tandem mass spectrometry (LC-MS/MS) measurement of 2-hydroxyethidium (2-OH-E^+^) and 2-hydroxymitoethidium (formed from DHE and MitoSOX, respectively) yields the only fully quantitative measurement of intracellular O_2_^•−^ generation [56,57]. The HPLC-based assay separates 2-OH-E^+^ from the non-specific oxidation product ethidium, allowing absolute calibration against authentic standards. Comparable approaches quantify boronate-probe phenol products, providing absolute H_2_O_2_ generation rates in cellular and tissue samples. Where available, MS-based quantification of probe products should be considered the most rigorous benchmark against which fluorescence-based methods are calibrated.

### 6.2. Oxidative-Damage Biomarkers

Beyond direct ROS measurement, MS plays a central role in oxidative-damage biomarker quantification. Isoprostanes—particularly 8-iso-prostaglandin F_2α_ (8-iso-PGF_2α_)—are formed by free-radical-catalyzed peroxidation of arachidonic acid in situ on phospholipids and represent the most reliable quantitative biomarker of in vivo lipid peroxidation [95,96]. Urinary isoprostane measurements have established population reference ranges, assessed oxidative stress in cardiovascular disease, smoking, pulmonary fibrosis, diabetes, and obesity, and have evaluated antioxidant interventions in clinical trials [95,96]. Quantification typically uses GC-MS or LC-MS/MS with deuterated internal standards.

Other validated MS-based biomarkers include 8-hydroxy-2′-deoxyguanosine (8-OHdG) and 8-oxo-7,8-dihydro-2′-deoxyguanosine (8-oxodG) for DNA oxidative damage, 3-nitrotyrosine for protein nitration by peroxynitrite, protein carbonyls for general protein oxidation, and 4-hydroxynonenal-protein adducts for lipid-derived electrophile damage [34,97,98]. Each biomarker requires careful sample preparation to avoid artifactual oxidation during processing—an issue that has plagued the field historically and that demands strict adherence to validated protocols [95,96].

### 6.3. Redox Proteomics

Redox proteomics—the systematic identification and quantification of cysteine oxidation states by MS—has matured into a powerful tool for mapping the cellular oxidative response [99,100]. Approaches include OxICAT (oxidant-isotope-coded affinity tags), iodoTMT (iodoacetyl tandem mass tags), and click-chemistry-based enrichment of sulfenic acids using dimedone-derived probes such as DYn-2 [97,98,99,100]. These methods enable proteome-wide identification of redox-modified targets, including discovery of physiological substrates of NOX-derived H_2_O_2_ signaling. Biologically important targets identified by redox proteomics include protein tyrosine phosphatases (PTP1B, PTEN), several transcription factors (NF-κB, AP-1, HIF-1α), metabolic enzymes (GAPDH, peroxiredoxin), and components of the autophagy machinery [97,98].

Recent developments include genetically encoded redox proximity probes that label proteins in proximity to a redox event in living cells, and chemoproteomic methods that identify the kinetic partners of specific redox modifications. The combination of MS-based redox proteomics with genetically encoded biosensors provides a uniquely powerful approach to dissecting compartmentalized redox signaling networks [11,98].

### 6.4. Redox Proximity Proteomics: Mapping Compartmentalized Signalling Networks

The most consequential conceptual shift in redox proteomics over the past five years is the recognition that compartmentalization of redox signalling cannot be resolved by global cysteine-oxidation surveys alone. The cellular thiol proteome contains tens of thousands of cysteines, only a small fraction of which are redox-active under physiological conditions; even fewer are causally linked to specific signalling outputs. Proximity-based labelling strategies, which mark proteins within nanometre distance of a defined redox event in living cells, address this problem directly and are emerging as the most promising approach for mapping the cause-and-effect architecture of redox signalling networks.

Three complementary platform classes have been developed. The first uses engineered ascorbate peroxidase (APEX, APEX2) or biotin ligase (TurboID, BioID2) fused to a redox-active reporter such as a peroxiredoxin or a redox-sensitive transcription factor; activation of the parent redox event triggers proximity-dependent biotinylation of nearby proteins, which are then enriched and identified by streptavidin pull-down and LC-MS/MS [99,100]. The second uses dimedone-derived sulfenic-acid reactive probes such as DYn-2 or its second-generation derivatives, which trap sulfenylated cysteines in living cells and enable quantitative mapping of the “sulfenome” under defined redox stimuli. The third, and the most recent, combines genetically encoded biosensors with proximity labelling: an APEX-HyPer fusion biotinylates proteins in proximity to a HyPer-detected H_2_O_2_ burst, allowing temporally and spatially resolved capture of the proteome that experiences a defined redox signal. This last class is the conceptual foundation for what we expect to become the standard mapping technology of compartmentalized redox signalling within this decade.

Several practical considerations apply. Proximity labelling produces a list of candidate interactors that includes constitutively biotinylated background proteins, abundant cytosolic species that diffuse through the labelling radius, and genuine proximity hits; rigorous use requires paired control conditions (catalytically inactive APEX, no-stimulus controls, isotope-labelling-based quantification) to subtract background and identify stimulus-specific changes. The labelling radius itself imposes a spatial-resolution limit: APEX-mediated tyrosine biotinylation operates over approximately 10–20 nm, while TurboID over a similar range; this is sufficient to resolve membrane microdomains and protein-complex composition but not individual subunits. The temporal resolution is set by the labelling time (typically 30 s to 10 min for APEX/TurboID activation), which is well matched to redox signalling kinetics. The output is a quantitative, organelle- and stimulus-specific protein interaction map that, in combination with HyPer7 or roGFP2-Orp1 spatiotemporal data, enables a mechanistic redox-signalling network rather than a static proteome inventory.

For the practising investigator, redox proximity proteomics should be regarded as the natural complement to compartment-resolved biosensor measurements: the biosensor reports when and where a redox event occurred; the proximity proteome reports which proteins were in physical and functional proximity. The two technologies in combination represent, in our view, the dominant mapping strategy for compartmentalized redox signalling for the next five to ten years, and we expect that disease-targeted applications—particularly in cancer, where redox signalling intersects with thioredoxin-system pharmacology, and in neurodegeneration, where compartment-specific redox dysfunction is increasingly recognised—will move from proof-of-concept to systematic deployment within this period.

## 7. Electrochemical Detection and Nanosensors

Electrochemical methods exploit the redox activity of ROS themselves, detecting changes in current at an electrode held at a defined potential. Hydrogen peroxide can be reduced or oxidized at platinum, gold, or modified carbon electrodes; superoxide is detected at cytochrome c-modified electrodes through electron-transfer reduction; and nitric oxide is selectively measured at platinized carbon-fiber sensors [101,102]. The principal advantage is real-time temporal resolution (millisecond to second range) without exogenous probes, avoiding the perturbation of the system being measured.

### 7.1. Macroelectrodes and Tissue Measurements

Macroelectrode-based approaches have been used for decades to monitor ROS efflux from cell suspensions and tissue preparations. Cytochrome c reduction by O_2_^•−^, read out spectrophotometrically at 550 nm, remains a standard technique for measuring NOX activity in isolated membranes and phagocyte preparations [102]. Continuous-flow Clark-type electrodes for H_2_O_2_ have been employed in perfused organ studies, providing temporal resolution that matches physiological events such as the respiratory burst and ischemia–reperfusion. The principal limitation of macroelectrode methods is poor spatial resolution; signals are averaged over the volume of the perfusate or sample.

### 7.2. Nanoelectrodes for Single-Cell Measurements

Nanoelectrodes—typically platinum or platinum-black-modified carbon fibers tapered to tip diameters of 100–500 nm—have enabled measurement of multiple ROS within single living cells [101]. By positioning the nanoelectrode adjacent to or within a single cell, investigators have demonstrated localized bursts of O_2_^•−^ and H_2_O_2_ release during phagocytosis, mitochondrial depolarization, and cell death [101,103]. The technique has been combined with patch-clamp electrophysiology to correlate redox events with membrane potential changes and with simultaneous fluorescence microscopy to provide multimodal characterization of single-cell redox dynamics.

Multiplexed sensing has resolved the relative contributions of H_2_O_2_, NO, ONOO^−^, and NO_2_^−^ to the overall reactive species output of activated immune cells, providing the first quantitative apportionment of these species in vivo [101]. Recent developments include dual-channel electrodes for simultaneous H_2_O_2_ and NO measurement, and microelectrode arrays for tissue-level mapping of redox heterogeneity. The foundational nanoelectrode studies cited above [101,102,103] have been substantially extended in the subsequent decade by primary work on platinized carbon, boron-doped diamond, MXene, and graphene-based platforms (see Section 7.3); the field’s earlier reliance on a small number of seminal demonstrations should not be misread as a static literature.

### 7.3. Nanomaterial-Based Sensors

Nanomaterial-based sensors using gold nanoparticles, graphene, carbon nanotubes, MXenes, or upconversion nanoparticles have been developed for both intracellular and in vivo ROS detection [104]. Such sensors offer enhanced sensitivity (often picomolar detection limits), the possibility of multimodal readout (electrochemical, fluorescent, photoacoustic, magnetic resonance), and surface chemistry that can be engineered for selectivity. In vivo nanosensor platforms have been demonstrated for tumor imaging, cardiac monitoring during ischemia, and inflammation tracking. Continued development of biocompatible coatings and selectivity layers is expected to extend electrochemical and nanosensor methods further into clinical and preclinical contexts [104,105].

Several specific platforms exemplify the current state of the art and warrant individual mention. Platinized carbon nanoelectrodes—produced by electrodeposition of platinum nanostructures onto pulled carbon-fiber tips—achieve detection limits of approximately 10–100 nM for H_2_O_2_ with millisecond temporal resolution and have been used to record real-time peroxide bursts from single mitochondria positioned at the electrode tip. Graphene-based flexible electrodes, conformable to soft tissue surfaces, have been deployed for continuous H_2_O_2_ monitoring on epicardial surfaces and skin wounds, with reproducibility maintained over 24–72 h windows. Boron-doped diamond microelectrodes provide an exceptionally wide potential window and resistance to organic fouling and have become the platform of choice for chronic in vivo measurements lasting days to weeks. Two- and three-dimensional MXene electrodes (Ti_3_C_2_T_x_ phases) and gold-nanoparticle-decorated graphene electrodes have reached picomolar detection limits for H_2_O_2_ in buffered solution under bench conditions, although sensitivity in complex biological matrices is typically one to two orders of magnitude lower because of fouling and competing electroactive species. Implantable wireless electrochemical patches that combine on-board signal conditioning, Bluetooth telemetry, and biofouling-resistant zwitterionic coatings have been demonstrated in rodent models for continuous monitoring of inflammation and oxidative stress over weeks [106,107].

### 7.4. Practical Limitations: Fouling, Calibration, and Stability

Three classes of practical limitation constrain the broader adoption of electrochemical and nanosensor methods, and any responsible application must address each. The first is electrode fouling. In any biological matrix containing protein, lipid, or fibrinogen, sustained polarization at oxidative potentials drives adsorption of macromolecules onto the electrode surface, with progressive attenuation of faradaic current that mimics or masks the redox signal of interest. Mitigation strategies include zwitterionic and poly(ethylene glycol) coatings that resist non-specific adsorption; Nafion overlayers that exclude anionic interferents and reduce protein adsorption; selective permselective membranes such as cellulose acetate; and electrochemical pre-treatment regimens (alternating positive–negative potential pulses) that periodically clean the surface during chronic recordings. None of these is a complete solution; in vivo sensors operating beyond approximately 72 h require either explicit drift correction (typically via parallel reference electrodes) or periodic in situ recalibration through co-implanted micro-reservoirs of a calibrating analyte.

The second limitation is calibration. Unlike fluorescent ratiometric biosensors, single-output amperometric and voltammetric sensors report a current that depends on probe concentration, mass transport, and electrode area, all of which can change during a measurement. Standard practice combines (i) bench calibration on standard solutions before and after the experiment; (ii) on-electrode microdosing of a calibration analyte during recording; and (iii) post-experiment ex vivo calibration of the explanted sensor. Where these are infeasible—for example in chronic implants—calibration drift is the principal source of systematic error and should be reported as an explicit uncertainty estimate alongside the primary signal. Selectivity calibration deserves equal attention: ascorbate, urate, dopamine, glutathione, and acetaminophen all give faradaic responses at potentials commonly used for H_2_O_2_ detection. Differential designs (sensor minus reference electrode functionalized identically except for the catalytic recognition layer) substantially reduce these interferences but do not eliminate them.

The third limitation is long-term stability. Catalytic surfaces lose activity through Pt restructuring, dissolution, and silver-chloride reference-electrode degradation; nanomaterial coatings can detach under shear or repeated potential cycling; and the foreign-body response in vivo encapsulates implanted electrodes in fibrotic tissue that progressively isolates them from the local extracellular fluid. The half-life of a typical chronic carbon-fiber sensor in cortex is on the order of weeks to a few months; for longer endpoints, replacement at scheduled intervals or self-renewing surface chemistries are required. Reported sensitivity should always be paired with the time interval over which it was measured: a sensor whose limit of detection is reported only at t = 0 in clean buffer cannot be assumed to retain that limit in vivo at t = 7 days. Standardized stability reporting (sensitivity at t = 0, t = 24 h, t = 7 d under realistic biological exposure) would substantially improve the cross-laboratory comparison of nanosensor platforms.

A particular caveat applies to the picomolar limits of detection routinely cited for MXene, gold-nanoparticle-on-graphene, and other advanced nanosensor platforms. These figures are almost always obtained in PBS or Krebs buffer with the analyte present at controlled concentration. In serum, blood, interstitial fluid, or wound exudate—the matrices in which clinical or chronic-preclinical measurements would actually be made—sensitivity collapses by one to two orders of magnitude, and selectivity degrades further because of competing electroactive species (ascorbate, urate, dopamine, glutathione, acetaminophen) and rapid biofouling. The trajectory of fouling is also non-linear: in serum-exposed sensors, an initial protein-conditioning layer forms within minutes, followed by a slower fibrinogen-and-platelet phase over hours, and a chronic foreign-body capsule over days to weeks. Each phase has a distinct effect on faradaic current and on diffusion to the electrode surface, and reporting only the t = 0 sensitivity in clean buffer is no longer adequate evidence that a nanosensor can serve as a chronic biological monitor. Mitigation strategies that have shown durable benefit in the recent literature include (i) zwitterionic phosphorylcholine, sulfobetaine, and carboxybetaine coatings that resist non-specific protein adsorption; (ii) hydrogel overlayers (alginate, PEG-based) that act as a diffusion-permissive but cell-excluding interface; (iii) Nafion or cellulose-acetate permselective membranes that reject anionic interferents while permitting H_2_O_2_ diffusion; and (iv) periodic in situ electrochemical pre-treatment that desorbs accumulated foulants. None of these is a complete solution; their combined application can extend functional in vivo half-life from days to several weeks, but the mismatch between bench-buffer detection limits and matrix-realistic detection limits remains a substantial gap that the field should report transparently. We recommend that nanosensor papers intended for biomedical application report, as a minimum, sensitivity in three matrices (PBS, 50% serum, and the intended biological compartment) at three time points (t = 0, 24 h, 7 d), so that the clinical-translational reader can assess realistic in vivo performance rather than only the bench peak.

Despite these constraints, electrochemical and nanosensor methods occupy a distinct and irreplaceable niche in the contemporary toolkit. Their unique combination of millisecond temporal resolution, real-time readout, and compatibility with chronic recording in awake animals cannot be matched by fluorescent, EPR, or MS approaches. As surface-engineering, microfabrication, and wireless telemetry continue to mature, electrochemical sensing is likely to become the principal platform for translational redox monitoring—a development that may, within this decade, deliver the first clinically deployed continuous biomarkers of oxidative stress in critical-care and chronic-disease settings.

## 8. Comparative Evaluation and Method Selection

No single method addresses all redox biology questions, and contemporary investigators are increasingly expected to apply complementary approaches and to validate findings across modalities [11,22]. The principal characteristics of the five platforms reviewed are summarized comparatively in Table 1, which extends the conventional sensitivity–selectivity–resolution rubric to include the practical decision criteria of full-pipeline implementation cost, required level of operator expertise, and risk of false-positive results—the three factors that, in our experience, most often determine the realistic deployability of a given platform in a particular laboratory.

### 8.1. Recommended Workflows

**Figure 3:**
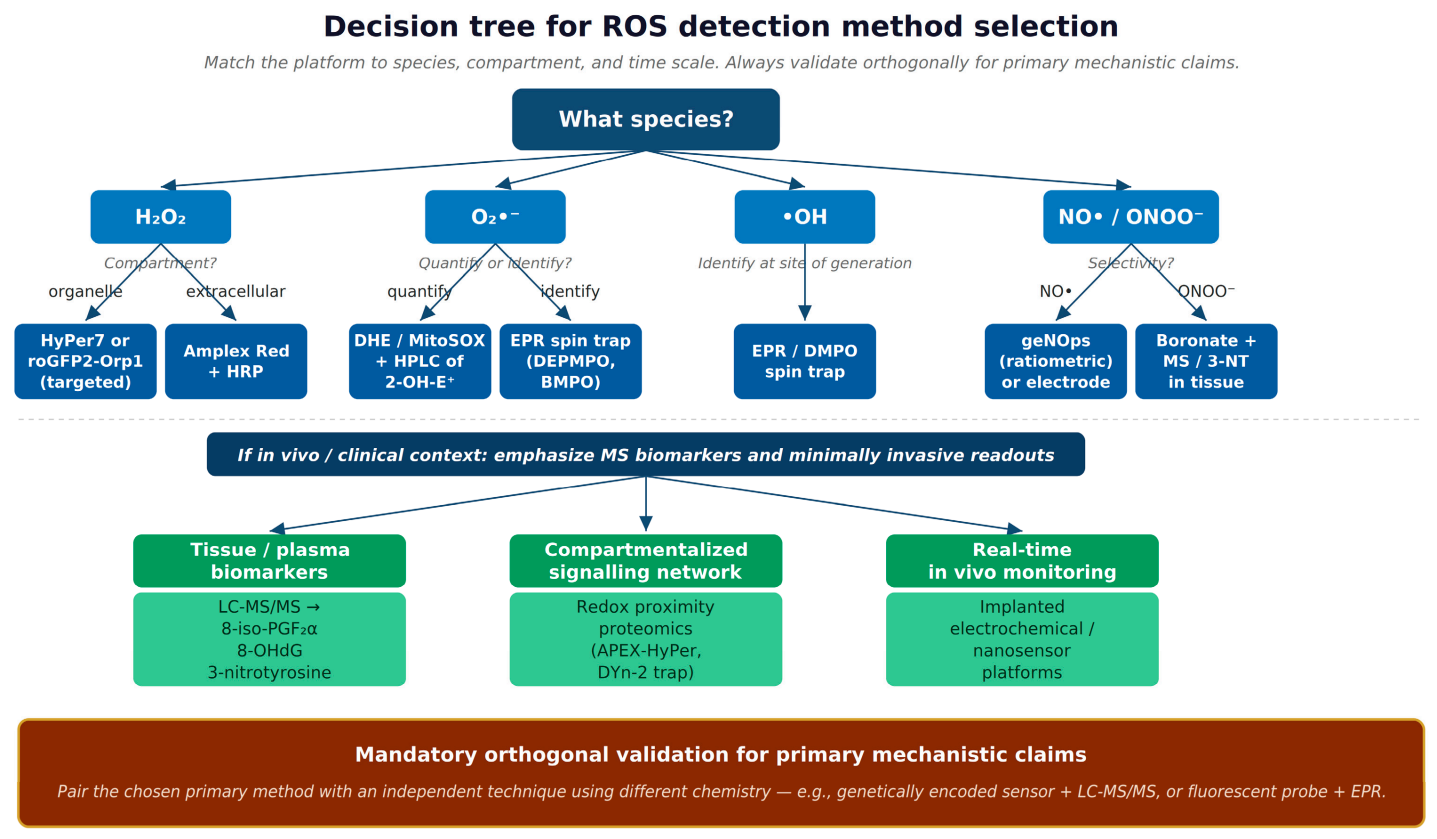
Decision tree for selecting an appropriate ROS detection method. The choice of platform is driven sequentially by (i) the chemical species of interest (H_2_O_2_, O_2_•^−^, •OH, NO•/ONOO^−^), (ii) the experimental compartment, time scale, and quantitative requirement, and (iii) the in vivo or in vitro context. Tissue and clinical applications privilege MS biomarkers and minimally invasive readouts. Across all branches, primary mechanistic claims require orthogonal validation by an independent technique using different chemistry (e.g., genetically encoded biosensor + LC-MS/MS, or fluorescent probe + EPR).

Several practical workflows can be recommended on the basis of the experimental question; the corresponding decision logic is summarized in Figure 3. (i) For compartment-specific H_2_O_2_ dynamics in living cells, the genetically encoded HyPer7 or roGFP2-Orp1, targeted to the compartment of interest with appropriate signal sequences, is the method of choice. Co-expression of SypHer or pH-sensitive controls is recommended. (ii) For identification of an unknown free radical in a mechanistic study, EPR spin trapping with DEPMPO or BMPO (for O_2_^•−^) or DMPO (for ^•^OH) provides unambiguous spectral identification. (iii) For quantitative O_2_^•−^ production rates in cellular preparations, DHE or MitoSOX combined with HPLC quantification of 2-OH-E^+^ is the gold standard.

(iv) For in vivo or clinical biomarker measurement, LC-MS/MS quantification of plasma or urinary 8-iso-PGF_2α_ (lipid peroxidation), 8-OHdG (DNA oxidation), and 3-nitrotyrosine (protein nitration), with deuterated internal standards, provides validated and reproducible readouts. (v) For redox proteomics, click-chemistry-based enrichment of sulfenic acids combined with quantitative mass-tag labeling identifies the proteome-wide consequences of a redox perturbation. (vi) For real-time single-cell measurements of multiple species simultaneously, electrochemical nanosensors offer unmatched temporal and chemical resolution.

In all cases, the cardinal principle is orthogonal validation: any conclusion drawn from a single method should, where possible, be confirmed by an independent approach with different chemistry [11,22]. A common and valuable combination is genetically encoded biosensors (for cellular dynamics) plus mass spectrometry (for absolute quantification and proteomic context).

### 8.2. Practical Probe-Selection Guide

Table 2 provides a working reference for investigators selecting a probe at the bench. The table lists representative starting conditions, spectral parameters, and one indispensable validation control per probe. The values given are typical starting points reported in the primary literature; final concentrations and incubation times should be optimized in the experimental system, and additional orthogonal controls (catalase, SOD, scavengers, parallel chemoselective probes, or LC-MS/MS quantification of probe products) should be applied as warranted by the rigour of the claim being made.

A small set of representative original studies illustrates each platform in practice and grounds the recommendations made here in primary evidence rather than expert opinion alone: quantitative EPR detection of cellular and mitochondrial superoxide with cyclic hydroxylamine spin probes [108]; ratiometric mass-spectrometric measurement of mitochondrial H_2_O_2_ in living animals with the MitoB exomarker, first in Drosophila and subsequently in mammalian (mtDNA-mutator mouse) tissue [109,110]; fluorescent boronate imaging of mitochondrial H_2_O_2_ with MitoPY1 [53]; and genetically encoded, ratiometric quantification of H_2_O_2_ with HyPer7 in mammalian cells and in vivo [75]. Together these exemplify the orthogonal, method-aware approach that this review advocates.

## 9. Limitations and Future Directions

Despite substantial methodological progress, important challenges remain. First, no single technique provides comprehensive ROS measurement; selecting the appropriate method requires careful matching of probe to species, compartment, time scale, and biological context [11,22]. Second, the absolute calibration of intracellular probe responses remains imperfect; quantitative claims of nanomolar H_2_O_2_ levels are based on indirect inference from kinetic models rather than direct calibration in living cells [11,15]. Third, in vivo translation of genetically encoded biosensors requires viral or transgenic delivery, limiting clinical applications to imaging modalities that do not require exogenous probes (such as photoacoustic detection of endogenous oxidation markers).

A scope limitation of this review should also be acknowledged. The methods surveyed are primarily those for detecting H_2_O_2_, O_2_^•−^, and •OH, with briefer treatment of HOCl, ^1^O_2_, and NO. Lipid-derived radical detection—EPR spin trapping with PBN-derivative or DMPO-based traps optimized for lipid radicals, fluorescent lipid-peroxidation sensors such as Liperfluo and its descendants, and the C11-BODIPY 581/591 ratiometric probe widely used in ferroptosis research—constitutes an important parallel toolkit that we now treat more directly: the fluorescent lipid-peroxidation probes (Liperfluo and the C11-BODIPY 581/591 ratiometric sensor) and their use in ferroptosis detection are discussed in a dedicated subsection (Section 4.6), while lipid-oxidation biomarkers (isoprostanes, MDA, 4-HNE) are quantified in the mass-spectrometry section. Given the centrality of lipid peroxidation to ferroptosis and to several disease contexts in which redox biology has advanced rapidly over the past five years, the reader should regard the present review as complementary to, not substitutive for, dedicated reviews of lipid-radical detection methods.

Looking beyond incremental probe development, several emerging analytical trends are likely to reshape redox measurement over the coming years and deserve fuller emphasis than they have so far received. Spatially resolved transcriptomics is beginning to map the expression of redox-regulatory and antioxidant genes within intact tissue architecture, allowing oxidative-stress signatures to be localised to specific cell populations and microenvironments rather than averaged across a dissociated sample [111]. In parallel, machine-learning and other artificial-intelligence methods are increasingly applied to redox data—from automated segmentation and denoising of biosensor images to the classification of oxidative-stress phenotypes and the prediction of redox-sensitive targets—offering a route to extract quantitative information from the large, noisy datasets that modern imaging and omics platforms generate [112]. Finally, multi-omics integration, in which mass-spectrometry-based redox proteomics (including reversible cysteine-oxidation mapping) is combined with transcriptomic, metabolomic, and lipidomic layers, promises to connect a measured oxidant signal to its downstream molecular consequences and to situate ROS detection within a systems-level description of redox biology [113]. These approaches remain at an early stage and are not yet routine, but their convergence with the validated detection platforms reviewed here is, in our view, among the most promising directions for moving the field from the measurement of bulk oxidant load toward spatially, temporally, and molecularly resolved redox physiology.

Three priorities should drive the field over the next decade. (i) Standardization: consensus protocols, reference materials, and reporting guidelines analogous to MIQE for qPCR are needed to improve reproducibility across laboratories. The American Heart Association consensus statement [22] and recent expert panels [3,11] provide useful starting points but lack universal adoption; a community-wide standard for reporting ROS measurements would allow meaningful comparison across studies and meta-analysis of redox-biology literature. (ii) Single-cell and spatial methods: integration of redox biosensors with single-cell omics, spatial transcriptomics, and intravital microscopy will reveal heterogeneity of redox states within tissues, organoids, and tumors at unprecedented resolution. (iii) Translational adoption: validated, scalable biomarkers—particularly MS-quantified isoprostanes, 8-OHdG, and protein-bound 3-nitrotyrosine—should be incorporated into clinical trials of redox-targeted therapeutics, replacing the still-prevalent reliance on the inadequate DCFH-DA assay [22,34,95].

Concrete examples illustrate that this is no longer aspirational. In cardiovascular disease, urinary 8-iso-prostaglandin F_2α_ measured by GC-MS or LC-MS/MS has been deployed as a stratification and pharmacodynamic biomarker in multiple controlled studies of statin and antioxidant therapy, in cardiac-surgery cohorts at risk of perioperative oxidative injury, and in heart-failure populations, with reproducible dose–response relationships and biological half-lives that support its use as a primary endpoint surrogate [95,96]. In pulmonary disease, 8-iso-PGF_2α_ in exhaled breath condensate and bronchoalveolar lavage has been employed as a quantitative pharmacodynamic readout in early-phase trials of antioxidant therapy in idiopathic pulmonary fibrosis, COPD, and acute respiratory distress syndrome, and as a stratification tool in cystic fibrosis. In oncology, 8-OHdG and 8-oxodG, quantified by isotope-dilution LC-MS/MS, have been used in early-phase studies of cancer chemoprevention and of redox-targeted therapeutics including thioredoxin-reductase inhibitors. In metabolic disease, isoprostane and 4-hydroxynonenal-protein-adduct measurements have informed phase I/II evaluation of mitochondrial-targeted antioxidants such as mitoquinone and SkQ1. The unifying lesson of these programmes is that MS-validated biomarkers, when measured with deuterated internal standards and standardized sample handling, deliver inter-laboratory variability comparable to routine clinical-chemistry analytes and are appropriate for use as primary or secondary endpoints in regulatory-grade trials. The remaining barrier is institutional rather than analytical: harmonized reference ranges, standardized pre-analytical protocols, and routine availability through clinical-laboratory networks would substantially accelerate adoption.

Emerging technologies that warrant attention include: super-resolution imaging combined with redox-responsive fluorophores, providing nanoscale localization of redox events; CRISPR-based genome-editing of endogenous loci to generate biosensor knock-in models that preserve native expression levels; activity-based proteomics using boronate-trap probes that capture redox-modified proteins for MS identification; photoacoustic imaging of endogenous melanin and hemoproteins as in vivo redox indicators; and machine-learning approaches that integrate multimodal redox data for prediction of cellular state. The next decade is likely to witness redox biology mature from a discipline focused on ROS as agents of damage to one focused on the spatiotemporal precision of redox signaling networks in health and disease.

## 10. Conclusions

The decade since the conceptual framework of oxidative eustress was formally articulated has witnessed a transformation in how ROS are measured in living systems. Boronate-based fluorescent probes, the HyPer7 and roGFP2-Orp1 genetically encoded biosensors, refined EPR spin-probe chemistry, redox proteomics by mass spectrometry, and electrochemical nanosensors have together enabled quantitative, compartment-resolved, and species-specific measurements that were inconceivable when DCFH-DA dominated the field. The biological understanding has advanced in parallel: H_2_O_2_ is now recognized as a regulated signaling molecule with nanomolar physiological concentrations, organelles maintain distinct redox set points, and disease states reflect compartment- and species-specific dysregulation rather than uniform oxidative excess.

For the practicing investigator, the implications are clear. Selection of an appropriate detection method must now be informed by the chemical species being measured, the cellular compartment of interest, the temporal scale of the process, and the requirement for quantitative versus qualitative claims. Reliance on a single non-specific probe should not be accepted as the primary or exclusive evidence for redox mechanisms in peer-reviewed publications. Orthogonal validation across complementary methods—typically a genetically encoded biosensor for cellular dynamics combined with mass spectrometry for absolute quantification—should become standard practice. For the field, the convergence of these methods promises a deeper mechanistic understanding of redox biology in health and disease, and the rational development of redox-targeted therapeutics that exploit, rather than indiscriminately suppress, the dual nature of ROS as both signal and damage agent [114].

## Figures and Tables

**Table 1:** Comparative evaluation of methods for ROS measurement in living systems.

Criterion	EPR	Small-Molecule Probes	Genetic Biosensors	Mass Spectrometry	Electrochemical/Nanosensors
**Direct radical detection**	Yes (gold standard)	Indirect (oxidation product)	Indirect (thiol oxidation)	Indirect (stable products)	Direct, real-time (amperometric) for O_2_^•−^ and H_2_O_2_
**Selectivity**	High (spectral fingerprint)	Variable (DCFH low; boronates high)	High (especially HyPer7, roGFP-Orp1)	High (*m*/*z* + retention time)	Moderate–high (electrode/membrane design)
**Sensitivity**	Modest (10^−7^–10^−9^ M)	High (sub-nM with Amplex Red)	Excellent (nM with HyPer7)	Excellent (sub-nM)	Excellent (pM–nM at nanoelectrodes)
**Spatial resolution**	Poor (mm in vivo)	Good with targeted probes	Excellent (organelle-resolved)	None (tissue average)	Good (single-cell with nanoelectrodes)
**Temporal resolution**	Seconds to minutes	Seconds (fluorescence)	Seconds (live imaging)	Endpoint only	Excellent (ms–s, real-time flux)
**Quantitative (absolute)**	Yes	No (relative only) without LC-MS	Semi-quantitative with calibration	Yes	Yes (calibrated current)
**Live-cell longitudinal**	Limited	Limited (probe loading, photobleaching)	Excellent	No (destructive)	Limited (electrode fouling; invasive)
**Cost/accessibility**	High instrument cost	Low (commercial reagents)	Moderate (transfection/animals)	High instrument cost	Low–moderate (potentiostat)
**Best application**	Radical identification, mechanism	Routine cellular ROS detection	Compartment-resolved live imaging	Biomarkers, redox proteomics	Real-time ROS flux at the cell surface/single cells
**Implementation cost (full pipeline)**	Very high (instrument €200k+; cryogenic accessories; specialist consumables)	Low–moderate (reagent kits €50–500; standard fluorescence instrumentation)	Moderate–high (transfection/AAV €100–2000 per construct; imaging instrumentation; transgenic-line maintenance)	Very high (LC-MS/MS instrument €300k–700k; isotope-labelled standards; expert sample preparation)	Low–moderate (potentiostat €5–30k; electrode fabrication)
**Required expertise level**	Specialist (EPR spectroscopy is rarely a generalist skill; trained physicist or chemist usually required)	Generalist (most cell biology laboratories can deploy with standard training)	Intermediate–advanced (molecular cloning, viral packaging, ratiometric quantification, image analysis)	Specialist (LC-MS/MS method development and quantitative redox proteomics require dedicated expertise)	Intermediate (electrochemistry/electrode fabrication)
**Risk of false-positive results**	Low (spectral fingerprint is diagnostic; principal risk is artefactual radical generation during sample preparation)	High for non-selective probes (e.g., DCFH-DA); moderate for boronate/chemoselective probes; low for HPLC-validated DHE 2-OH-E^+^ readouts	Low–moderate (provided pH/photobleaching controls are included and orthogonal validation is performed)	Low (provided sample handling avoids artefactual oxidation; deuterated internal standards essential)	Moderate (electroactive interferents; surface fouling)

**Table 2:** Practical probe-selection guide for ROS measurement in living systems.

Probe/Sensor	Target ROS	Typical Loading/Concentration	Excitation/Emission (nm)	Key Validation Control
DCFH-DA	Bulk oxidants (non-selective)	5–25 µM, 30 min in serum-free medium	488/525	Catalase (1000 U/mL) quench; parallel chemoselective probe
Boronate probes (PF1, PG1, PC1)	H_2_O_2_ (peroxynitrite cross-reactivity)	5–10 µM, 30–60 min	488/510 (PG1); variable	Catalase quench; control reaction with H_2_O_2_ standard
MitoPY1	Mitochondrial H_2_O_2_	5 µM, 30 min	510/540	FCCP collapse of ΔΨm to abolish mitochondrial accumulation
DHE/MitoSOX	O_2_^•−^	DHE 5–10 µM; MitoSOX 1–5 µM, 15–30 min	Excitation: 396 (2-OH-E^+^) vs. 480 (ethidium); emission ~580	HPLC separation of 2-OH-E^+^ from ethidium; SOD or Mito-TEMPO suppression
Amplex Red/HRP	Extracellular H_2_O_2_	50–100 µM Amplex Red + 0.1 U/mL HRP	563/587	Catalase quench; H_2_O_2_ standard curve
HyPer7	Cytosolic/organellar H_2_O_2_	Genetically expressed (transfection or AAV)	Ratiometric: 420/500 → emission 516	SypHer or pHRed-mt as internal pH control; DTT/H_2_O_2_ end-point calibration
roGFP2-Orp1/Tsa2ΔCR	H_2_O_2_ (via peroxiredoxin relay)	Genetically expressed	Ratiometric: 405/488 → emission 510	DTT (fully reduced) and diamide (fully oxidized) reference points
DMPO/DEPMPO/BMPO (EPR spin traps)	O_2_^•−^ and •OH	10–50 mM in cell or tissue suspension	EPR spectrum (X-band)	SOD-quenchable signal for O_2_•^−^; DMSO scavenge for •OH
MitoB/MitoP	Mitochondrial H_2_O_2_ (LC-MS/MS endpoint)	50–200 nM systemic dosing	LC-MS/MS quantification of MitoP	MitoB/MitoP ratio normalization; isotope-labelled internal standard

## Data Availability

The original contributions presented in this study are included in the article. Further inquiries can be directed to the corresponding author(s).

## Abbreviations

The following abbreviations are used in this manuscript:

ABS	activity-based sensing
APF	aminophenyl-fluorescein
ARE	antioxidant response element
BMPO	5-tert-butoxycarbonyl-5-methyl-1-pyrroline-N-oxide
CAT	catalase
CMH	1-hydroxy-3-methoxycarbonyl-2,2,5,5-tetramethylpyrrolidine
CPH	1-hydroxy-3-carboxy-2,2,5,5-tetramethylpyrrolidine
cpYFP	circularly permuted yellow fluorescent protein
DCFH-DA	2′,7′-dichlorofluorescin diacetate
DCF	2′,7′-dichlorofluorescein
DEPMPO	5-(diethoxyphosphoryl)-5-methyl-1-pyrroline-N-oxide
DHE	dihydroethidium (hydroethidine)
DMPO	5,5-dimethyl-1-pyrroline-N-oxide
DUOX	dual oxidase
eNOS	endothelial nitric oxide synthase
EPR	electron paramagnetic resonance
ER	endoplasmic reticulum
FRET	Förster resonance energy transfer
GFP	green fluorescent protein
GPx	glutathione peroxidase
Grx	glutaredoxin
GSH	reduced glutathione
HOCl	hypochlorous acid
HPLC	high-performance liquid chromatography
Keap1	Kelch-like ECH-associated protein 1
LC-MS/MS	liquid chromatography–tandem mass spectrometry
MnSOD	manganese superoxide dismutase
MS	mass spectrometry
NADPH	nicotinamide adenine dinucleotide phosphate
NO	nitric oxide
NOX	NADPH oxidase
Nrf2	nuclear factor erythroid 2-related factor 2
ONOO−	peroxynitrite
PCL-1	Peroxy Caged Luciferin-1
PG1	Peroxy Green-1
PF1	Peroxyfluor-1
8-iso-PGF2α	8-iso-prostaglandin F2α
Prx	peroxiredoxin
roGFP	reduction-oxidation-sensitive green fluorescent protein
RNS	reactive nitrogen species
ROS	reactive oxygen species
RONS	reactive oxygen and nitrogen species
SOD	superoxide dismutase
SOSG	singlet oxygen sensor green
TPP+	triphenylphosphonium cation
Trx	thioredoxin
